# Stress, senescence, and specialized metabolites in bryophytes

**DOI:** 10.1093/jxb/erac085

**Published:** 2022-03-08

**Authors:** Samarth Kulshrestha, Rubina Jibran, John W van Klink, Yanfei Zhou, David A Brummell, Nick W Albert, Kathy E Schwinn, David Chagné, Marco Landi, John L Bowman, Kevin M Davies

**Affiliations:** The New Zealand Institute for Plant and Food Research Limited, Private Bag 11600, Palmerston North 4442, New Zealand; The New Zealand Institute for Plant and Food Research Limited, Private Bag 92169, Auckland Mail Centre, Auckland 1142, New Zealand; The New Zealand Institute for Plant and Food Research Limited, Department of Chemistry, Otago University, Dunedin, New Zealand; The New Zealand Institute for Plant and Food Research Limited, Private Bag 11600, Palmerston North 4442, New Zealand; The New Zealand Institute for Plant and Food Research Limited, Private Bag 11600, Palmerston North 4442, New Zealand; The New Zealand Institute for Plant and Food Research Limited, Private Bag 11600, Palmerston North 4442, New Zealand; The New Zealand Institute for Plant and Food Research Limited, Private Bag 11600, Palmerston North 4442, New Zealand; The New Zealand Institute for Plant and Food Research Limited, Private Bag 11600, Palmerston North 4442, New Zealand; Department of Agriculture, Food and Environment, University of Pisa, Italy; School of Biological Sciences, Monash University, Melbourne, VIC, Australia; The New Zealand Institute for Plant and Food Research Limited, Private Bag 11600, Palmerston North 4442, New Zealand; University of Parma, Italy

**Keywords:** Flavonoid, hornwort, liverwort, moss, phenolic, terpenoid

## Abstract

Life on land exposes plants to varied abiotic and biotic environmental stresses. These environmental drivers contributed to a large expansion of metabolic capabilities during land plant evolution and species diversification. In this review we summarize knowledge on how the specialized metabolite pathways of bryophytes may contribute to stress tolerance capabilities. Bryophytes are the non-tracheophyte land plant group (comprising the hornworts, liverworts, and mosses) and rapidly diversified following the colonization of land. Mosses and liverworts have as wide a distribution as flowering plants with regard to available environments, able to grow in polar regions through to hot desert landscapes. Yet in contrast to flowering plants, for which the biosynthetic pathways, transcriptional regulation, and compound function of stress tolerance-related metabolite pathways have been extensively characterized, it is only recently that similar data have become available for bryophytes. The bryophyte data are compared with those available for angiosperms, including examining how the differing plant forms of bryophytes and angiosperms may influence specialized metabolite diversity and function. The involvement of stress-induced specialized metabolites in senescence and nutrient response pathways is also discussed.

## Teaser

We review how specialized metabolites help plants cope with the abiotic and biotic stresses of the terrestrial environment, and compare emerging knowledge on bryophytes with that available for angiosperms.

## Introduction

Life on land exposes plants to many environmental stresses not faced by aquatic plant species (de Vries and Archibald, 2018; Fürst-Jansen *et al.*, 2020). A key evolutionary change thought to have occurred during the transition from life in water to life on land, 515–470 million years ago, is a marked expansion in metabolic capabilities (Weng *et al.*, 2012; Weng, 2014; Fürst-Jansen *et al.*, 2020). This is particularly so for the pathways that produce ‘secondary’ or ‘specialized’ metabolites—compounds with specific functions outside the core ‘primary’ metabolic pathways common to many plant cells. These specialized metabolites, which are often stress-induced, can assist with tolerance to abiotic stresses, such as fluctuations in visible light and temperature, UVB-radiation exposure, and drought, as well as to biotic challenges from pathogens, pests, and plant competitors (Chen *et al.*, 2018; Davies *et al.*, 2018; Ferreyra *et al.*, 2021; Horn *et al.*, 2021). Comparative genomic studies across the major groups of extant land plants—bryophytes, lycophytes, ferns, and seed plants—indicate that core components of several major specialized metabolite pathways are present across land plants but absent from extant algal relatives (Yonekura-Sakakibara *et al.*, 2019; Davies *et al.*, 2020; de Vries *et al.*, 2021). This suggests that they arose during land colonization and were present in an early common ancestor of extant land plants but not in the algal-like predecessors of land plants.

Subsequent to land colonization, the expansion of available ecological niches is thought to have promoted ongoing evolutionary diversification of specialized metabolite pathways. This is most apparent in angiosperms, with a massive expansion of species diversity over relatively recent evolution, but is also expected to be the case for those plants that rapidly diversified following colonization of land—the bryophytes. Bryophytes are the non-tracheophyte land plant group, comprising the Anthocerotophyta (hornworts, about 300 species), Marchantiophyta (liverworts, about 9000 species), and Bryophyta (mosses, about 12 000 species). Mosses and liverworts show a similar distribution to flowering plants with regard to available environments, able to grow in polar regions through to hot desert landscapes, and are the predominant plant species of Antarctica.

The initial biosynthetic steps that convert amino acids to the key precursors of the major specialized metabolite pathways, such as those for phenolic and terpenoid metabolism, appear conserved across land plants (Yonekura-Sakakibara *et al.*, 2019; Davies *et al.*, 2020; de Vries *et al.*, 2021. Patterns of gene expression in response to environmental triggers also seem well conserved. Subsequent diversification of metabolic capacity from the initial pathway compounds has resulted from gene duplication and neo-functionalization of biosynthetic enzymes, as well as diversification of the transcription factors regulating the temporal and spatial specificity of biosynthetic gene expression (Weng, 2014; Catarino *et al.*, 2016; Bowman *et al.*, 2017; Fürst-Jansen *et al.*, 2020). This has been studied in detail for angiosperms but only more recently for bryophytes. Early studies suggest that in bryophytes comparatively less diversification of transcription factors has occurred. However, there has been substantial diversification of biosynthetic activities, including generation of larger gene numbers for some enzyme types than has been found in angiosperms (Chen *et al.*, 2018; Davies *et al.*, 2020; de Vries *et al.*, 2021). For the bryophytes examined to date, often the ‘core’ genes of the early steps from amino acids to major pathway precursors are present as single genes or small gene families, while the gene families that generate specific compound variations later in the biosynthetic pathways have diversified between genera or species (Bowman *et al.*, 2017; Davies *et al.*, 2020; Zhang *et al.*, 2020; de Vries *et al.*, 2021). This can provide the basis for each species having a unique compound profile, including the many metabolites reported, to date, only from bryophytes.

In this article we summarize knowledge on the comparative occurrence of specific specialized metabolite pathways in bryophytes compared with angiosperms. There are extensive data on the phytochemistry of bryophytes. However, genetic or physiological data are mostly from a limited set of ‘model’ species—in particular the moss *Physcomitrium patens* and the complex thalloid liverwort *Marchantia polymorpha*. Yet, the dominant bryophyte species in many ecosystems are quite different from the model species—such as the *Sphagnum* mosses and leafy liverworts. We examine how the differing life cycles and plant forms of bryophytes and angiosperms may influence diversity of specialized metabolites and the timing and localization of their production, including consideration of diverse bryophyte forms. The involvement of specialized metabolites in senescence and nutrient response pathways is also discussed—an underexplored aspect of bryophyte genetics and physiology. We finish with brief coverage of how climate change may affect niche distribution of bryophyte species, particularly how this may alter the contribution of specialized metabolites to maintaining ecosystems.

## Specialized metabolite pathways of bryophytes

Data on bryophyte chemistry is relatively limited across species diversity because of practical difficulties in collecting pure samples of morphologically small plants (often present as species mixes), the challenges of taxonomic identification, and a lack of research funding compared with economic crops. However, there are extensive data on the phytochemistry of some bryophyte species that support chemotaxonomy or have been motivated by potential agrochemical or human medical bioactive applications of the extracted compounds. Bryophytes have featured in traditional medicinal practices, with around 70 species having established uses (Harris, 2008; Asakawa *et al.*, 2013*a*, *b*; Martínez-Abaigar and Núñez-Olivera, 2021; Drobnik and Stebel, 2021). Bryophyte extracts have been demonstrated to be active against plant and human microbial pathogens; plant fungal pathogens and insect pests; and as allelopathic compounds (Asakawa *et al.*, 2013*a*, *b*; Commisso *et al.*, 2021). In contrast, only recently have the *in planta* functions of the metabolites started to be demonstrated.

Studies to date have identified bryophyte compounds belonging to the metabolite classes of phenolics, terpenoids, and alkaloids, the same classes that account for the great majority of specialized metabolites in angiosperms (Table 1) (Asakawa *et al.*, 2013*a*, *b*). However, the relative prevalence of these is quite different between the plant groups; for example, there is extensive terpenoid diversity in bryophytes but a limited occurrence of alkaloids. Furthermore, within each of the classes, specific biosynthetic branches may be present only in one plant group, rather than being ubiquitous. More commonly, reflecting the associated extensive species diversity, there are angiosperm compounds yet to be reported for bryophytes. However, there are also examples of biosynthetic pathways specific to liverworts, mosses, or hornworts. In the next section we give an overview of bryophyte stress-related specialized metabolites, focusing on phenolics (particularly flavonoids) as the most relevant to abiotic stress tolerance and senescence pathways.

**Table 1.** Occurrence of selected specialized metabolite types in bryophytes and angiosperms, with an emphasis on phenolic compounds

Compounds	Hornworts	Liverworts	Mosses	Angiosperms
Terpenoids				
Monoterpenes	Common	Common	Common	Common
Diterpenoids	Common	Common	Common	Common
Sesquiterpenoids	Common	Common	Common	Common
Carotenoids	Common	Common	Common	Common
Alkaloids	Rare	Rare	Rare	Common
Phenylpropanoids				
Coumarins	Not reported	Common	Common	Common
Rosmarinic acid	Common	Not reported	Not reported	Yes
Lignans and/or neolignans	Common	Yes	Yes	Common
Anthocerotonic acid type neolignans	Common	Not reported	Not reported	Not reported
Stilbenes, e.g. resveratrol	Not reported	Yes	Not reported	Yes
Bibenzyls	Not reported	Common	Yes	Yes
* *Bisbibenzyls	Not reported	Common	Not reported	Yes
Phenanthrenes^*a*^	Not reported	Yes	Yes	Yes
Aurones	Not reported	Yes	Rare^*b*^	Yes
Auronidins	Not reported	Common	Not reported	Yes^*c*^
Flavones	Not reported	Common	Common	Common
Biflavonoids/triflavonoids	Not reported	Rare	Common	Yes
Flavonols	Not reported	Not reported	Common	Common
Proanthocyanidins	Not reported	Not reported	Not reported	Common
3-Deoxyanthocyanins	Not reported	Not reported	Yes	Yes
Anthocyanins	Not reported	Not reported	Not reported	Common
Sphagnorubins	Not reported	Not reported	Yes	Not reported
Other phenolics				
Hydrolysable tannins	Not reported	Not reported	Not reported	Common
Naphthalenes	Not reported	Yes	Yes	Common
Phenolamides/phenylamides	Not reported	Not reported	Not reported	Common

^*a*^ Predicted biosynthetic route involving bibenzyls, based on structure and co-occurrence with bibenzyls in liverworts and orchids.

^*b*^ Aurone-flavanone biflavonoids (Geiger and Markham, 1992) and a single aurone report (Weitz and Ikan, 1977).

^*c*^ Single report of cell culture compound (Taniguchi *et al.*, 2000).

Comprehensive reviews of data on metabolite occurrences in bryophytes are available in Asakawa *et al.* (2013*a, b*), and the reader is referred to these for details of individual compounds within the major metabolite classes.

## Terpenoids

Extensive reviews are available of bryophyte phytochemistry (Asakawa *et al.*, 2013*a*, *b*; Martínez-Abaigar and Núñez-Olivera, 2021; Commisso *et al.*, 2021). Table 1 presents a summary of the major compound types reported for angiosperms, liverworts, mosses, and hornworts. The majority of studies have been conducted on liverworts, with very few on hornworts. Bryophytes are enormously rich in terpenoid diversity, with more than 2000 structures reported (Xie and Lou, 2009; Asakawa *et al.*, 2013*a*, *b*; Chen *et al.*, 2018; Martínez-Abaigar and Núñez-Olivera, 2021). Bryophytes contain ‘typical’ plant terpene synthase genes, but also a class of microbial-like terpene synthase genes not present in angiosperms that produce bryophyte-specific terpenoids (Jia *et al.*, 2016; Chen *et al.*, 2018; Zhou and Pichersky, 2020). These microbial-like terpene synthase genes are thought to result from multiple independent horizontal gene transfer events that have occurred in non-seed land plants, but not in seed plants. Notable features of bryophyte terpenoids include the presence of many enantiomers of known angiosperm compounds; the richness of triterpenoids in mosses; and the great diversity of terpenoid structures in liverworts. Liverworts have been studied more extensively than other bryophytes, but the abundance and diversity of terpenoids in these species also may reflect the biological functions of liverwort oil bodies. Oil bodies are membrane-bound specialized organelles that originate from the fusion of secretory vesicles. They are present in abundance in many liverwort species and often accumulate terpenoids, tocopherol, tocopherol derivatives, and bibenzyls. Our understanding of the formation and function of oil bodies has advanced recently, with the identification of the class I homeodomain leucine-zipper (C1HDZ) and ERF/AP2-type transcription factors as key promoters of oil body differentiation and the genes required for secretion of compounds into the bodies (Kanazawa *et al.*, 2020; Romani *et al.*, 2020).

**Fig. 1.**
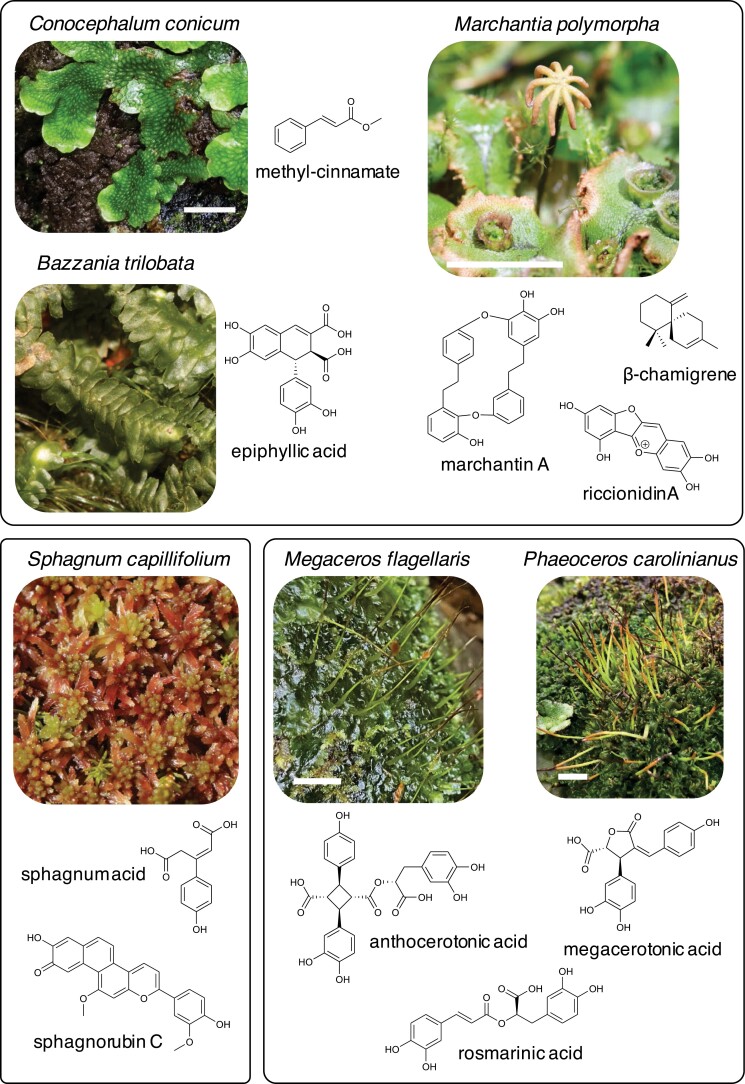
Examples of specialized metabolites discussed in this article, with images of species known to produce them. Top panel shows two thalloid and one leafy (*Bazzania trilobata*) liverwort species, the lower left panel shows the moss *Sphagnum capillifolium*, and the lower right panel shows two hornwort species. Scale bar (where shown) represents 1 cm. All photographs by the authors except for *B. trilobata* and *S. capillifolium*, which are WikimediaCommons/HermannSchachner (https://commons.wikimedia.org/wiki/File:Bazzania_trilobata_(a,_144632-474722)_6252.jpg; https://commons.wikimedia.org/wiki/File:Sphagnum_capillifolium_(c,_141136-472352)_0994.JPG, accessed 5 November 2021; both licensed under CC1.0).

In general, assigning specific functions to such a large and diverse range of compounds is one of the major future challenges for research on terpenoids in bryophytes. Terpenoids contribute to many of the phytochemical characteristics of liverworts—such as the distinctive scents and pungency plus bitterness if tasted (Asakawa *et al.*, 2013*a*, *b*). Liverworts can emit scents (e.g. *Corsinia coriandrina*, which has a coriander-like scent, and *Conocephalum* spp.), especially if damaged. In addition to containing terpenoids, the scents can also contain simple alcohols and phenolics (the biosynthesis of which is discussed in the next section). Cinnamic acid derivatives in particular are important volatiles of liverworts, with methyl-cinnamate an illustrative example, being produced in abundance in ‘great scented liverworts’ (*Conocephalum* spp.; Fig. 1). This compound is also associated with angiosperm floral scent. The key enzyme, CINNAMIC ACID-METHYLTRANSFERASE, has been characterized in both bryophytes and angiosperms, and phylogenetic comparisons suggest independent convergent evolution (Zhang *et al.*, 2019). Volatile function in liverworts is not well defined, but methyl-cinnamate has been suggested to be antimicrobial.

## Phenolics

Phenolics are the second largest group of specialized metabolites in bryophytes, with reported compound diversity only a little smaller than that for terpenoids (Asakawa *et al.*, 2013*a*, *b*). Phenolics are also of greater significance in terms of compound abundance, since they are produced both as bioactives and in large amounts as structural components of the cell wall and for the formation of polymers of the cuticle. Relatively simple cinnamic acid derivatives originating from the first few steps of the phenylpropanoid pathway are present in all land plants. Some types, such as coumarins, appear to be ubiquitous. Others are only sporadically produced in angiosperms but are abundant in some bryophytes, notably rosmarinic acid (Fig. 1), which may comprise >5% dry weight in cell cultures of the hornwort *Anthoceros agrestis* (Vogelsang *et al.*, 2006). Some phenylpropanoids are produced in taxonomically specific patterns. With respect to bryophytes, notable examples are the bibenzyls/bisbibenzyls of liverworts and the lignans of hornworts. Bibenzyls are produced by only a small number of angiosperm taxa, but are widespread and diverse in structure in liverworts. They are also the precursors of more complex derivatives that are not present in angiosperms, such as the cyclic bisbibenzyls (e.g. marchantin A in Fig. 1) and cannabinoid-like compounds (Asakawa *et al.*, 2013*a*, *b*; Hussain *et al.*, 2018). For *M. polymorpha*, there has been good progress in identifying some of the candidate biosynthetic enzymes and genes, with R2R3MYB *Mp*MYB02 having been identified as the direct transcriptional activator of the pathway (Kubo *et al.*, 2018). Lignans and neolignans, dimers of cinnamic acid derivatives with or without a β–β linkage, respectively, are abundant in hornworts and include structures not reported from other plants (e.g. anthocerotonic acid and megacerotonic acid in Fig. 1). Polymeric phenolics are a characteristic feature of bryophytes, including polymeric flavonoids in mosses. However, lignin polymers have not been reported, reflecting their non-tracheophyte plant status.

## Flavonoids

The flavonoid pathway differs significantly between angiosperms and bryophytes and between each bryophyte group with regard to the classes of flavonoids produced. Several major flavonoid classes are absent from bryophytes, and hornworts appear to completely lack flavonoids (Asakawa *et al.*, 2013*b*). The reason for the lack of flavonoids in hornworts is unknown. It is possible the ancestor of hornworts diverged from the ancestor of other land plants prior to the evolution of the flavonoid pathway. As the prevalent theory is that bryophytes are monophyletic (Leebens-Mack *et al.*, 2019), it is more probable that a hornwort ancestor acquired mutations in flavonoid biosynthetic and/or regulatory genes (Yonekura-Sakakibara *et al.*, 2019; Davies *et al.*, 2020, 2021). This implies that either hornworts evolved alternative mechanisms that fulfil the functions of flavonoids or at some point the hornwort ancestor was in an environment in which selection pressure was low for retention of flavonoid functions.

Liverworts have the flavonoid biosynthetic pathway leading to flavanones and flavones, including the yellow aurones, but lack the enzyme FLAVANONE 3-HYDROXYLASE that is required to form the dihydroflavonol precursors of flavonols, proanthocyanidins and anthocyanins (D. D. Li *et al.*, 2020). In addition to containing most flavonoid classes found in liverworts, mosses also produce dihydroflavonols and flavonols, and some produce 3-deoxyanthocyanins. However, like other bryophytes, mosses lack proanthocyanidins and the 3-hydroxyanthocyanins prevalent in angiosperms. There are additional compound classes with more taxonomically limited production in angiosperms, such as isoflavonoids, that are also missing from bryophytes.

Most phenylpropanoids present in bryophytes are also found in angiosperms. However, liverworts and mosses contain classes of red flavonoid pigment that are essentially absent from angiosperms, specifically auronidins and sphagnorubins, respectively (Fig. 1). These are both cell wall-bound pigments, the functional significance of which is discussed later. The biosynthetic pathway to auronidins via aurones in *M. polymorpha* has been partially characterized (Berland *et al.*, 2019), and the key transcriptional activator identified as the R2R3MYB protein encoded by Mp*MYB14* (Albert *et al.*, 2018; Kubo *et al.*, 2018). The biosynthetic pathway to sphagnorubins, which structurally are 3-deoxyanthocyanidins with two additional aromatic rings connected to the A-ring (Berland and Andersen, 2021), is unknown. The genetics and evolution of the phenylpropanoid biosynthetic pathway have been reviewed recently (Yonekura-Sakakibara *et al.*, 2019; Davies *et al.*, 2020; Piatkowski *et al.*, 2020; de Vries *et al.*, 2021). Thus, these areas are not addressed in detail here. Despite excellent recent progress, there are many outstanding research questions concerning polyphenolic biosynthesis in bryophytes. These include the reason for the lack of flavonoids in hornworts; how flavonoids are produced in mosses in the absence of CHALCONE ISOMERASE (CHI); the biosynthetic route to create sphagnorubins and 3-deoxyanthocyanins in mosses; biosynthetic intermediates, transport mechanisms, polymerization status, and functions of auronidins and sphagnorubins in cell walls; biosynthetic pathways for the phenolics that play key roles in *Sphagnum* bog ecology; and regulation of the flavonoid pathway in mosses.

## Other specialized metabolites

Specialized metabolites containing nitrogen or sulphur are rare in bryophytes (Asakawa *et al.*, 2013*a*, *b*). This is in contrast to angiosperms, which contain several major classes of alkaloids, with more than 12 000 reported structures (Xie and Lou, 2009; Gutiérrez-Grijalva *et al.*, 2020). Among the few reported compounds from bryophytes, notable examples include the nitrogen- and sulphur-containing glucosinolates from liverworts (von Reuß and König, 2005), the alkaloid anthocerodiazonin produced by *A. agrestis* (Trennheuser *et al.*, 1994), and violet-red aminochrome pigments from *M*. *polymorpha* (Busch *et al.*, 2019).

Despite the progress in characterizing phytochemistry, knowledge on the specialized metabolite pathways of bryophytes is far behind that for angiosperms. Analysis is particularly limited with regard to species and tissue diversity, seasonality, and environmental conditions. For example, in a recent publication on plant natural product discovery, a survey of literature found new compounds reported from 165 plant species, but only three of the species examined were bryophytes (Lautié *et al.*, 2020). There are also geographical regions from which information on bryophyte species is limited or absent. Moreover, information on the biosynthetic pathways is available for just a few compounds, such as specific terpenoids and flavonoids, and much of this has been obtained only recently. It cannot be assumed that biosynthetic pathways characterized in angiosperms apply to bryophytes, as similar compounds may arise from different routes; for example, how the phenolic precursors for cuticle polymers are produced (Renault *et al.*, 2017). The contribution of associated microbial partners to bryophyte phytochemistry, such as fungal endophytes of liverworts or the hornwort–cyanobacterium symbiosis (Frangedakis *et al.*, 2021), is only now starting to be explored. One of the most notable knowledge gaps is the limited understanding of *in planta* functions of the specialized metabolites in bryophytes. In the next section we examine some possible functions with regard to abiotic and biotic stress. There have been recent reviews or comprehensive articles for specific metabolite groups in this regard (Chen *et al.*, 2018; Davies *et al.*, 2018; Zhou and Pichersky, 2020). Subjects featured in those reviews are covered only briefly here, to highlight the most recent advances. To augment this, we address in more detail how specialized metabolite functions may reflect bryophyte growth habits and the modification of their local environment, including nutrient responses and senescence pathways.

## Specialized metabolites and stress tolerance in bryophytes

Bryophytes face many of the same environmental stresses as other land plants—excess photosynthetically active radiation (PAR), UVB-radiation exposure, fluctuations in temperature, drought, and attacks by pathogens and pests. Given that liverworts and mosses occupy some of the most extreme environments of any plants, including Antarctica and hot deserts, they have developed effective stress tolerance mechanisms. Bryophytes and angiosperms share some stress-tolerance pathways thought to be inherited from the common ancestor of land plants. However, the distinct plant forms and lifestyles of bryophytes may also have given rise to unique stress-tolerance mechanisms. Although underground tuber formation has been reported for liverworts such as *Fossombronia* living in semi-arid environments (Stotler *et al.*, 2003), bryophytes lack the complex bulbs, corms, and tubers that can vegetatively maintain angiosperms through periods of environmental challenge. However, many bryophyte species have desiccation-tolerant vegetative growth, which enables survival through less favourable times. Additionally, they produce spores as a sexual reproductive unit analogous to seeds, and some have gemmae as clonal propagules for maintaining individuals through times of stress.

Many mosses and leafy liverworts have a growth pattern in which the tissues behind the meristem enter a programmed senescence pathway. Where this occurs, only the apical part of the plant remains photosynthetic, the proximal region is alive but non-photosynthetic, and the distal region is senescing or fully decomposed. Water and nutrients accumulate in, and are transported up from, the region of dead plant material. This is dramatic in *Sphagnum* bogs, where the photosynthetic top part of the plant may be growing on decaying material, slowly transforming into peat that is several metres deep. Phenolics are a key component of the ecology of *Sphagnum* bogs, modifying the local physical environment and microbiome, and contributing much of the dry matter to peat.

The initial biosynthetic steps within the major specialized metabolite pathways are thought to have evolved in a common ancestor of all land plants. Also, the different pathways seem to retain at least some common physiological functions in nearly all land plant groups. Terpenoids are important angiosperm defence compounds against pathogens and pests. Flavonoids have been shown to contribute across all stages of the angiosperm lifecycle to tolerance to stress, including UVB-radiation, drought, fluctuations in PAR and temperature, quenching of reactive oxygen species (ROS), and protection against biotic attack. There is now evidence for all these metabolite functions in at least some bryophytes. Although there is a lack of controlled studies, researchers have observed that liverworts and mosses show less damage from pathogens and pests than angiosperms, despite lacking the physical defences of the woody vascular plants, suggesting they contain effective anti-pathogen compounds. Indeed, there have been a great many studies demonstrating bioactivity against plant pathogens and pests of extracts or pure compounds from liverworts, and to a lesser extent mosses (Asakawa *et al.*, 2013*a*, *b*; Commisso *et al.*, 2021). Thus, it is reasonable to suggest that the purpose of many of the terpenoids, bibenzyls, and other specialized metabolites is plant defence. However, demonstration of *in planta* functionality is generally lacking, with most data coming from recent studies on *M. polymorpha*. The importance of oil bodies, and the associated specialized metabolites, to biotic defence in *M. polymorpha* was demonstrated using lines with mutations in either the Mp*C1HDZ* or ERF/AP2-like transcription factor genes that normally promote oil body differentiation (Kanazawa *et al.*, 2020; Romani *et al.*, 2020). Loss-of-function Mp*c1hdz* lines had reduced expression of specialized metabolism genes and depletion of oil body terpenoids. Transcript abundance for the activator of bisbibenzyl biosynthesis, Mp*MYB02*, was greatly reduced in Mp*c1hdz* lines, but there was no difference in the expression of Mp*MYB14*. The Mp*c1hdz* lines had lower amounts of monoterpenes, sesquiterpenes, and bisbibenzyls, and their extracts had reduced antibacterial activity. However, although the extracts, as with extracts of many other liverwort species, demonstrated anti-microbial activity *in vitro*, the *in planta* studies support a function in anti-herbivory. In the case of the Mp*c1hdz* lines, the Crustacean *Armadillidium vulgare* (known as the pill bug, slater, roly-poly bug, or common woodlouse) had a preference for feeding on mutant plants lacking oil bodies rather than the wild-type (Kanazawa *et al.*, 2020; Romani *et al.*, 2020). Similarly, *M. polymorpha* plants with reduced terpenoid production because of mutations in the Mp*MYCY* gene involved in jasmonate signalling were more susceptible to herbivory by *Spodoptera litoralis* (Peñuelas *et al.*, 2019).

Model systems have been established for infection of *M. polymorpha* by oomycete (*Phytophthora palmivora*), fungal (*Colletotrichum* sp.), and bacterial (*Pseudomonas syringae*) pathogens (Nelson *et al.*, 2018; Carella *et al.*, 2019). A notable response to *P*. *palmivora* infection is the localized production of auronidin, controlled by Mp*MYB14* (Carella *et al.*, 2019). Induction of auronidin production by ectopic overexpression of Mp*MYB14* reduced hyphal penetration and infection rates, while auronidin-lacking Mp*myb14* mutants displayed enhanced disease susceptibility compared with wild-type plants. Colonization of *Marchantia paleacea* or *M. polymorpha* by endophytic fungi also induced strong red cell wall pigmentation (Humphreys *et al.*, 2010; Nelson *et al.*, 2018), and a recent study on infection of *M. polymorpha* with the vascular wilt fungal pathogen *Fusarium oxysporum* found induction of Mp*MYB14* and phenylpropanoid pathway genes (Redkar *et al.*, 2021, Preprint). Thus, auronidin deposition in the cell wall is a conserved part of the response to microbial interactions for at least some liverworts. A function in preventing microbial infection matches the sites of highest auronidin production, which are where tissues are in contact with the soil—in the ventral scales and the ventral midrib of the thallus. Whether auronidin is directly antimicrobial or can be incorporated into a polymer that reduces cell wall digestibility has yet to be established. However, the resistance of auronidin to solvent extraction *in vivo* indicates strong cell wall association and a function in wall strengthening.

Several pathogenic microbes have been shown to infect *P*. *patens*, including bacteria, fungi, and oomycetes. As observed for *M. polymorpha*, infection of *P*. *patens* with some of these pathogens induced flavonoid pathway gene expression and accumulation of phenolics in the cell wall (Ponce de León and Montesano, 2013, 2017; Otero-Blanca *et al.*, 2021), although the specific phenolics produced were not characterized. Lignans are one possibility, but in *Sphagnum* species many oxidative derivatives of sphagnum and cinnamic acids are found bound to the cell wall. A recent comprehensive transcriptome analysis following *Colletotrichum gloeosporioides* infection of *P*. *patens* found multiple specialized metabolite pathways to be induced, with phenylpropanoid-related genes among the most strongly activated (Otero-Blanca *et al.*, 2021).

Although the mechanisms have not been extensively explored, there are many studies that show inhibitory activities of liverwort specialized metabolites against bryophyte spore germination or angiosperm seed germination and/or root growth (Basile *et al.*, 2003; Asakawa *et al.*, 2013*a*; Wang *et al.*, 2013; Whitehead *et al.*, 2018; Liu *et al.*, 2020). Allelopathy associated with these compounds could be an important factor for discouraging the establishment of vascular plants that would later shade bryophytes, and also for establishing balances of plant species and microbiomes within bryophyte communities. This could be of particular significance within complex bog ecosystems composed of different *Sphagnum* species. Studies have found extensive evidence of allelopathy influencing the structure of the bog microbial community (Chiapusio *et al.*, 2018; Hamard *et al.*, 2019). There is also recent evidence for phenolics having an allelopathic function between plant species (Liu *et al.*, 2020), and for some volatile compounds enabling communication between *Sphagnum* species (Vicherová *et al.*, 2020).

Many of the biosynthetic pathways that produce anti-pathogen compounds also produce compounds for tolerance to abiotic stress. These can be the same compounds, such as in the case of some antioxidants and, potentially the cell wall-bound pigments. The most extensive data on functions of specialized metabolites in association with abiotic stress tolerance in bryophytes are for responses to the light environment—specifically excess UVB radiation or PAR. The core UVB-radiation protection mechanism of UV RESISTANCE LOCUS8 (UVR8)-induced flavonoid production is well conserved between *M. polymorpha* and angiosperms (Clayton *et al.*, 2018; Kondou *et al.*, 2019). This includes UVB perception by UVR8, transcriptional changes mediated by ELONGATED HYPOCOTYL5 (HY5), and feedback repression by REPRESSOR OF UVB PHOTOMORPHOGENESIS (RUP) (Clayton *et al.*, 2018). Evidence suggests this system also operates in mosses (Newsham *et al.*, 2005; Clarke and Robinson, 2008; Wolf *et al.*, 2010; Waterman *et al*., 2017, 2018; Soriano *et al.*, 2018*b*; C. Li *et al.*, 2019; Ferreyra *et al.*, 2021), although definitive data from moss mutant lines missing specific phenolic classes are lacking. The same flavonoid compounds are key for UVB-radiation tolerance in *M. polymorpha* and angiosperms—flavones and flavones/flavonols, respectively (Clayton *et al.*, 2018; Soriano *et al.*, 2018*a*). However, other compounds may also have more significant roles in mosses, such as biflavonoids or cell wall-bound phenolics, including sphagnorubins (Newsham *et al.*, 2005; Clarke and Robinson, 2008; Waterman *et al.*, 2017, 2018; Ruklani *et al.*, 2021). There are very limited data available for hornworts, but cell wall-bound phenolics may also be important in this bryophyte group (Soriano *et al.*, 2018*b*). The *A. agrestis* genome does contain sequences encoding the key response components UVR8, HY5, and RUP (F. W. Li *et al.*, 2020; Zhang *et al.*, 2020; and the authors’ analysis), but there are no data on what their downstream biosynthetic pathway targets might be.

Many liverwort and moss species have been shown to respond to excess PAR by producing red flavonoid pigments (Hooijmaijers and Gould, 2007; Waterman *et al.*, 2018; Berland *et al.*, 2019). To date, these have been found to be the cell wall-bound auronidins and sphagnorubins in liverworts and mosses, respectively. Mosses can also produce 3-deoxyanthocyanins. This was first reported 60 years ago (Bendz *et al.*, 1962), but there are no data on *in planta* functions of 3-deoxyanthocyanins in mosses. In contrast to sphagnorubins, which are aglycones with poor water solubility and are tightly associated with the cell wall (Berland and Andersen, 2021), the 3-deoxyanthocyanins are glycosylated and so have higher water solubility and, potentially, could accumulate in the vacuole in a similar manner to the 3-hydroxyanthocyanins of angiosperms. The absence of the 3-hydroxy shifts the absorption maxima to a shorter wavelength and the associated colour range towards orange-red. However, in ferns at least, 3-deoxyanthocyanins provide red coloration in foliage similar to that from 3-hydroxyanthocyanins in angiosperms (Cohen *et al.*, 2002; Davies *et al.*, 2020). Some mosses also produce colourless cell wall-bound phenolics in response to PAR-related stress (Clarke and Robinson, 2008). The stress tolerance functions have not been explored, but most phenolic acids are able to absorb UVB radiation. No data are yet available for hornworts on stress tolerance mechanisms for excess PAR.

How the same phenolic compounds provide tolerance benefits to such a variety of different stresses is not yet clear in bryophytes, or indeed in angiosperms, nor is it known why pigmentation is so variable within plant groups, such as among *Sphagnum* species. In angiosperms anthocyanins are induced by many different biotic and abiotic triggers; can be accumulated specifically in different tissues, individual cell layers, or subcellular compartments; and can vary in patterns of secondary modification dependent on the perceived stress (Kovinich *et al.*, 2014; Landi *et al.*, 2015; Davies *et al.*, 2018). Whether anthocyanins have a universal function in photoprotection during different stress events (as light screens and/or antioxidants) or have specific functions in responses to the individual stresses is unresolved (Landi *et al.*, 2015; Gould *et al.*, 2018; Renner and Zohner, 2019; Agati *et al.*, 2020; Lo Piccolo *et al.*, 2020; Z. C. Yu *et al.*, 2021; Hughes *et al.*, 2022). For example, the function of increased anthocyanin production in autumn leaves is still vigorously debated (Agati *et al.*, 2021; Pena-Novas and Archetti, 2021, and references therein). Auronidins are produced by different liverwort species in a range of tissues and different stress situations, including in photosynthetic parts of leafy liverworts under PAR stress (Hooijmaijers and Gould, 2007); in the purple-black ventral scales that cover the enrolled thallus in desiccation-tolerant *Riccia* species; in the long purple scales that hang from the undersurface of floating *Ricciocarpos natans* (Davies *et al.*, 2020); and in *M. polymorpha* in response to nitrogen or phosphate starvation (Albert *et al.*, 2018; Kubo *et al.*, 2018; Rico-Reséndiz *et al.*, 2020) or pathogen attack (Carella *et al.*, 2019). Definitive functional data are lacking for nearly all examples of auronidin or sphagnorubin production, and this is a significant gap in our knowledge of flavonoid biology.

An abiotic stress that can be deleterious for bryophytes living in unfavourable environments is deficiency in various nutrients. Nutrient supply often presents different challenges to bryophytes than to angiosperms, reflecting growth habits that may limit access to any nutrients that are beyond the surface region of soil or rocks. Thus, bryophytes may have evolved different mechanisms to influence external nutrient supply, or to remobilize nutrients from within the plant. Specialized metabolites have significant functions in some of the known examples of these processes. Some bryophytes die following spore production, and in species such as *M. polymorpha* the green gemmae can remain on plants that have otherwise undergone general senescence, patterns analogous to seed or tuber production in angiosperms. However, there are few data on the associated bryophyte senescence pathways. Perhaps the best characterized example is *Sphagnum* phenolic production and programmed tissue senescence that contributes to establishing a plant and microbial community that favours moss-growing conditions, including nutrient status, and discourages the establishment of other plant species. The bog environment is unfavourable to the microbes that decompose dead plant material in soil, and *Sphagnum* contributes substantially to this in many peat bogs as an ‘ecosystem engineer’ (Bacon *et al.*, 2017). The non-photosynthetic hyaline cells that make up most of the plant volume encourage water retention, which given the common locations of peat bogs, presents a cold, water-saturated, and acidic environment. These cells also influence the bog ecosystem in two important ways through their cell wall chemistry, which is rich in unique carbohydrates and polyphenolics, the latter of which are excreted into the bog as monomers. The pectin-like sphagnan (galacturonic acid-based) carbohydrate portion has cationic properties that lead to bog water acidification through sequestration of both nitrogen (e.g. NH_4_^+^) and positively charged micronutrients (e.g. Mg^2+^ and Ca^2+^). The sphagnans also contribute to the formation of the supramolecular humic acids through conjugation with amino acids and proteins (Verhoeven and Liefveld, 1997; Hájek *et al.*, 2011). Additionally, senesced *Sphagnum* material is resistant to microbial attack, probably because of the accumulation of simple phenolic monomers, principally sphagnum acid, which is generally considered the most abundant phenolic produced (Fig. 1), and more complex polyphenolics, including flavonoid glycosides (Verhoeven and Liefveld, 1997; Fudyma *et al.*, 2019). *Sphagnum* also releases specialized metabolites that are directly antimicrobial (Hamard *et al.*, 2019). Together, these factors contribute to formation of a nutrient-poor and largely anoxic environment. The anaerobic conditions have been proposed to limit activity of oxygen-dependent enzymes that degrade phenolics, such as phenoloxidases. The resulting accumulation of phenolics might then bind and inhibit other degradative enzymes, as well as binding and sequestering substrates and having direct antimicrobial activities (Verhoeven and Liefveld, 1997). This mechanism of inhibiting microbially mediated tissue decomposition has been termed the ‘enzyme latch’ (Freeman *et al.*, 2001). However, the enzyme latch theory and functional significance of phenolics in the bog environment remains a subject of debate (McGivern *et al.*, 2021; Urbanová and Hájek, 2021).

## Senescence processes in bryophytes and the roles of specialized metabolites

Senescence is the final stage of the lifecycle of a cell, tissue, or organism. It can be triggered by the gradual decline in physiological functions during ageing, which may be accelerated under stress, but in plants is often a programmed developmental stage (Woo *et al.*, 2018). It can also be a variable response (ranging from single cell or tissue to a whole organism), which under unfavourable environmental conditions or to contain biotic attack allows reallocation of resource to reproductive tissues. Study of plant senescence requires integration of data on environmental conditions, seasonality, and intrinsic regulatory pathways (Lihavainen *et al.*, 2021; Sultana *et al.*, 2021; Xue *et al.*, 2021; Zhang *et al.*, 2021).

Senescence contributes to an optimized growth pattern in a given ecological habitat. For angiosperms, this may range from the annual loss of leaves in deciduous trees, to the seasonal death of above-ground organs in species with subterranean storage (e.g. bulbs, corms, and tubers), to the monocarpic flowering and seed distribution of annuals. The role of senescence in bryophytes is much less studied, but notable examples include the previously mentioned progressive senescence of *Sphagnum* mosses and mat-forming leafy liverworts, and also the rapid sexual- and vegetative-reproductive strategies of species occupying short-lived habitats such as slips in unstable riverbanks. The continuous senescence gradient presented in erect-growing, mat-forming mosses and leafy liverworts could provide an outstanding system for deciphering the senescence pathways of bryophytes, and the contribution of specialized metabolite production to ecosystem maintenance. Recent studies have started to address in more detail how differences in the decomposition rate of different *Sphagnum* species can help establish niche differentiation along microhabitat gradients (Piatkowski *et al.*, 2021).

**Fig. 2.**
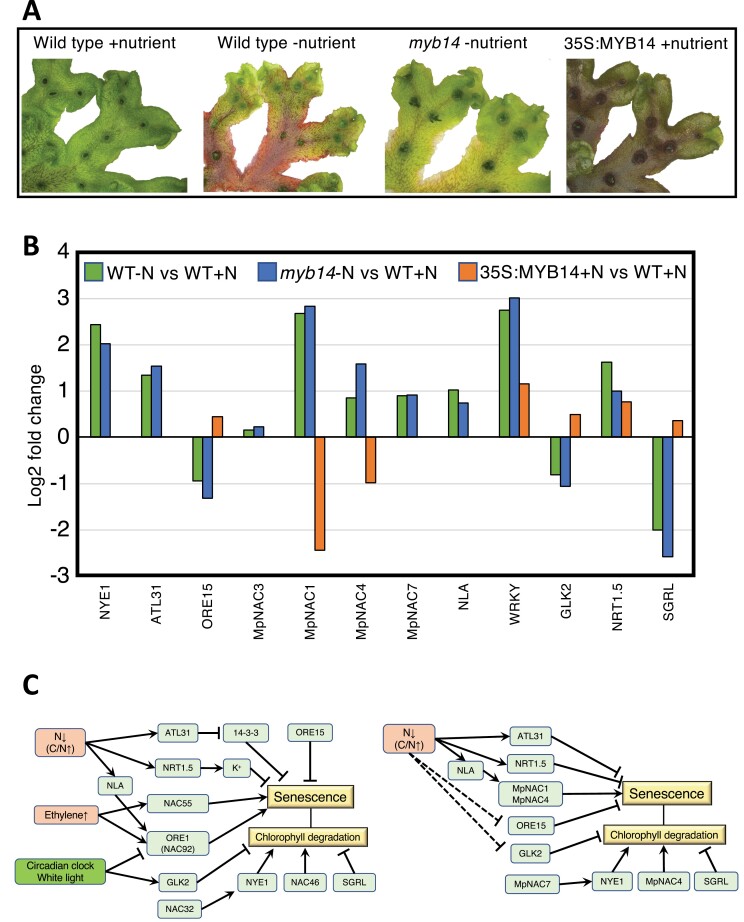
Comparison of senescence pathways in *Marchantia polymorpha* and Arabidopsis. (A) Phenotypes of *M. polymorpha* showing senescence induced by nutrient deficiency. From left to right are wild-type lines on full (+nutrient) or minimal (−nutrient) medium, a line with a mutation for *myb14* on minimal medium, and a *35S:MYB14* overexpression line on full medium. The experimental conditions are reported in Albert *et al.* (2018) and plants shown are approximately 6 weeks old. (B) Transcript abundance values for candidate senescence-associated *M. polymorpha* genes in differential expression comparisons between the lines shown in (A), from the RNAseq data of Berland *et al.* (2019). WT, wild-type. The *M. polymorpha* genes are those with a close sequence match to known senescence-associated genes of Arabidopsis (tblastn with an E-value of <10^−5^) that also have RNAseq DEseq2 scores of adjusted *P*<0.001. Gene IDs for the *M. polymorpha* genes, and possible Arabidopsis equivalents where indicated, are: NYE1, AtNYE1/AtSGR/Mapoly0001s0049/Mp1g17090; ATL31, AtATL31/Mapoly0002s0161/Mp1g27170; ORE15, AtORE15/Mapoly0006s0089/Mp3g06190; NAC3, MpNAC3/Mapoly0011s0176;Mp4g11910; NAC1, MpNAC1/AtORE1/Mapoly0015s0058;Mp2g07720; NAC4, MpNAC4/AtNAC46/Mapoly0020s0051/Mp4g22890; NAC7, MpNAC7/AtNAC32/GRAB1-like/Mapoly0035s0049/Mp6g02620; NLA, AtNLA/Mapoly0044s0127/Mp4g0346; WRKY, MpWRKY7/Mapoly0039s0030/Mp3g17660 (there was no single clear Arabidopsis WRKY match); GLK2, AtGLK2/ MpGARP8/Mapoly0156s0007/Mp7g09740; NRT1.5, AtNRT1.5/Mapoly0204s0004/Mp8g09440; SGRL, AtSGRL/Mapoly0113s0009/Mp1g02610. (C) Simplified summary of the functions of the Arabidopsis genes in promoting or delaying leaf senescence (left) and those genes that showed a conserved pattern of gene response in *M. polymorpha* under nutrient deficiency (right).

Setting aside the localized hypersensitivity response to pathogen infection, the genetic control of senescence is best understood for leaves. Localized senescence either as a developmentally programmed part of ageing or as a stress response may enable remobilization of nutrients from the affected region, principally from the chloroplast where the majority of nitrogen in leaves is located, to provide better chances of plant survival and an increased tolerance capacity in surviving tissues. It commonly involves changes to cell structure, degradation of macromolecules, and resorption or mobilization of nutrients such as nitrogen, phosphorus, potassium, and sulphur (Guiboileau *et al.*, 2010; Havé *et al.*, 2017; Masclaux-Daubresse *et al.*, 2017; Aguera and De la Haba, 2018; Sade *et al.*, 2018; Wen *et al.*, 2020; Lihavainen *et al.*, 2021). Most data on genetic pathways for developmentally regulated senescence in plants have been generated from studies of aged Arabidopsis leaves and the flag leaves that are crucial to the seed development of grain cereals (Havé *et al.*, 2017; Sultana *et al.*, 2021). Nitrogen deficiency affects the C/N balance, accelerating senescence in many angiosperms. It induces disruptions in several core processes, including photosynthetic efficiency, source–sink relationships, and hormone balances. These result from interconnected effects of nitrogen shortage on metabolism and its role as a signalling molecule in the C/N balance, for example altering the phosphate starvation response pathway and the production of hormones such as abscisic acid (Guiboileau *et al.*, 2010; Sade *et al.*, 2018; Medici *et al.*, 2019; Wen *et al.*, 2020; Lihavainen *et al.*, 2021). The central regulator of age-dependent leaf senescence in Arabidopsis is the NAC transcription factor ORESARA (ORE1, ANAC092) (Woo *et al.*, 2004; Kim *et al.*, 2018; Y. C. Yu *et al.*, 2021) (Fig. 2). The principal link of ORE1 to signals on nitrogen status is through the NITROGEN LIMITATION ADAPTATION (NLA) protein, with disruptions of *NLA* gene transcription altering the timing of leaf senescence. NLA directly regulates ORE1 by protein–protein interactions that affect ORE1 stability through ubiquitin-mediated degradation (Park *et al.*, 2018). *NLA* transcript abundance is in turn regulated, at least in part, through targeting by *miR827*, the abundance of which is elevated under phosphate-deficient conditions and decreased by nitrogen starvation. Induction of anthocyanin production is characteristic for plants experiencing nitrogen- and/or phosphate-deficient conditions in a range of angiosperm species. This production of anthocyanins can delay plant senescence during nitrogen deficiency (Liang and He, 2018; Wen *et al.*, 2020), perhaps by maintaining a higher C/N ratio, which we discuss in more detail later. The At*nla* mutant under nitrogen deficiency does not show anthocyanin induction, and the mutants exhibit early senescence (Peng *et al.*, 2008). The nitrogen- and phosphate-response pathways in Arabidopsis regulate anthocyanin production via DELLA and SPX4 regulator-mediated changes in production of the key anthocyanin pathway transcriptional activators, the R2R3MYBs PRODUCTION OF ANTHOCYANIN PIGMENT1 (PAP1) and PAP2 and the bHLH TRANSPARENT TESTA8 (TT8) (Zhang *et al.*, 2017; He *et al.*, 2021). There are many physiological studies on autumnal leaf senescence in deciduous species, but few studies on associated gene expression changes (Song *et al.*, 2021). How stress alters senescence-related gene expression, and the functions of specialized metabolite pathways in this are relatively poorly described. A recent comparison of gene expression profiles in acyanic versus cyanic senescing leaves is one of the few studies available (Vangelisti *et al.*, 2020).

There are few genetic or physiological data for senescence processes in bryophytes. What exists is limited to preliminary studies on general signalling pathways or information on specific cells undergoing programmed senescence, such as mucilage or stomatal cells (Renzaglia *et al.*, 2017). Potential regulatory genes have been identified relating to some hormone pathways, specifically for abscisic acid-induced senescence in *P. patens* (Li *et al.*, 2018) and jasmonate responses in *M. polymorpha* (Monte *et al.*, 2019). Nitrogen or phosphate starvation of *M. polymorpha* has been shown to induce flavonoid pigmentation via R2R3MYB-mediated gene activation (Albert *et al.*, 2018; Kubo *et al.*, 2018; Rico-Reséndiz *et al.*, 2020). This is a characteristic response to nitrogen shortage in many angiosperms, but whether senescence-related genes are also induced has not been examined. However, homologues of key senescence pathway genes are present in the three major groups of bryophytes, suggesting the regulatory pathway is conserved across land plants (e.g. Fig. 2). To investigate the function of candidate homologues further, we examined the RNAseq data of Berland *et al.* (2019) for the response of candidate genes in *M. polymorpha* exposed to nutrient stress. The experiment from which the RNAseq data were obtained compared wild-type plants to lines with a knockout mutation for *Mp*MYB14, the transcription factor activating production of the red auronidin pigments, grown on complete or minimal media. Berland *et al.* (2019) also presented RNAseq data for *35S:MpMYB14* overexpressor plants (Albert *et al.*, 2018) that have strong, constitutive production of auronidins. Transcript abundance for the *M. polymorpha* genes closest in sequence conservation to At*ORE15*, At*NLA*, At*NAC1*, and At*NAC46*, which promote senescence in Arabidopsis, was higher in both the wild-type and Mp*myb14* mutant plants grown under nitrogen-deficient conditions (Fig. 2, which also provides gene IDs). Both sets of plants also had increased transcript abundance for candidate homologues to genes involved in chloroplast degeneration (*NYE1* and *GLK2*) and the responses to disrupted C/N balance (*ATL13*). Although the Mp*myb14* mutation did not markedly change the gene response compared with wild-type under nitrogen-deficient conditions, transcript abundance was lower for Mp*NAC1* and Mp*NAC4* and higher for Mp*ORE15* and Mp*GLK2* in *35S:MpMYB14* plants compared with wild-type plants when both were grown on full medium. This supports a link between production of flavonoids and the inhibition of senescence pathways in *M. polymorpha*, indicative of additional conservation of flavonoid functions in abiotic stress tolerance between liverworts and angiosperms.

As mentioned earlier, production of auronidin or sphagnorubin can be induced by a similar range of stresses as anthocyanins in angiosperms, including nutrient stress or tissue senescence. However, as the only consensus on functions of anthocyanins in angiosperm plant–environment interactions is that they have diversity of function, it is problematic to extend functional data on soluble, vacuolar anthocyanins of angiosperms to the cell wall-bound auronidins and sphagnorubins. Even when comparative studies of wild-type plants to mutant lines lacking production of specific flavonoids have demonstrated a phenotypic consequence, the underlying mechanism is still debated. However, the different chemical characteristics of anthocyanins relative to auronidins/sphagnorubins and the variation in plant forms and lifestyles between angiosperms and bryophytes may indicate what properties and functions of red flavonoid pigments are common across land plants. For example, there are two major theories for the functions of anthocyanin production in leaves of deciduous trees in autumn—‘photoprotection/photomodulation’ and ‘coevolution’ (Agati *et al.*, 2021; Pena-Novas and Archetti, 2021). In the first, anthocyanins facilitate photoprotection by direct light screening and/or as ROS scavengers to protect the photosystem. One consequence of maintaining the photosystem function longer during leaf senescence can be an improved resorption of nutrients, benefiting new growth in the spring. In the second theory, anthocyanin production is proposed to have coevolved with insect pests as an honest warning signal of chemical defences. Most recently, there has been a proposal that combines components of both these theories, linking them to influences from soil nutrient characteristics (Hughes *et al.*, 2022).

Strong PAR and cold stress together, as common in autumn, are among the most effective environmental inducers of anthocyanin biosynthesis and photoinhibition. Increased PAR can also induce auronidin or sphagnorubin production (e.g. Hooijmaijers and Gould, 2007; Bonnett *et al.*, 2010; Albert *et al.*, 2018; Waterman *et al.*, 2018), and increased red coloration of *Sphagnum* bogs in autumn is commonly observed. Anthocyanins, auronidins, and sphagnorubins are all optimally suited as effective shields against supernumerary photons, and so provide photoprotection, although whether auronidins and sphagnorubins are produced in cells appropriate to providing a photoprotective function has not been examined. Many studies have demonstrated higher photoprotection in anthocyanic compared with non-anthocyanic leaves (for a comprehensive discussion see Agati *et al.*, 2021), and in the few studies conducted to date on liverworts and mosses, red tissues also had improved photoprotection compared with green tissues (Hooijmaijers and Gould, 2007; Waterman *et al.*, 2018). Thus, anthocyanins, auronidins, and sphagnorubins may have a shared function in direct screening of PAR, regardless of their differing structures, mobility, and cellular localization.

It is more difficult to present an equally credible conserved function for the three types of red pigment in plant–pest communication, such as would underpin a common coevolution theme. It is also perhaps more difficult to propose functions for cell wall-bound auronidins and sphagnorubins in modulating stress-induced alterations in cellular redox homeostasis, as has been proposed for anthocyanins (e.g. Page *et al.*, 2012; Viola *et al.*, 2016). However, the biosynthetic intermediates prior to the cell wall-bound forms are unknown and could possibly have antioxidant properties. Nor can it be ruled out that each pigment type has specific functionalities, despite the common induction triggers. It is also possible they share common mechanism(s) in photoprotection but have additional functionalities in each case, such as cell wall strengthening by auronidins and sphagnorubins and ROS scavenging by anthocyanins.

Production of specialized metabolites can account for a significant proportion of carbon fixed in specific tissues. One proposal for how anthocyanins provide broad stress tolerance benefits is as a carbon sink or ‘sugar buffer’ for maintenance of C/N balance during periods of carbon excess or nitrogen limitation (Grace and Logan, 2000; Hernandez and Van Breusegem, 2010; Gould *et al.*, 2018; Soubeyrand *et al.*, 2018; Lo Piccolo *et al.*, 2020). The diversion of malonate from photoassimilation pathways directly into flavonoids could reduce localized carbon excess, without the nitrogen requirement associated with protein biosynthesis. Such C/N imbalances may occur when photosynthetic processes are disrupted by abiotic stress, such as during autumn in temperate zones. They can be a trigger for the onset of senescence, as well as being a central component of leaf senescence during the recovery of nitrogen from chloroplasts (Krieger-Liszkay *et al.*, 2019; Lo Piccolo *et al.*, 2020; Wen *et al.*, 2020; Lihavainen *et al.*, 2021). Such a mechanism could encompass auronidins and sphagnorubins, but is challenged by the nitrogen-containing red betalain pigments that are ‘anthocyanin replacements’ in some species of the Caryophyllales (Davies *et al.*, 2018; G. Li *et al.*, 2019).

## Climate change, specialized metabolites, and bryophytes

Rising average temperatures and more severe periods of drought are both predicted from the prevalent global climate change models. Both can generate an array of changes to habitats that increase plant abiotic stress, and these may be particularly severe for environments such as bogs and the polar regions in which bryophytes are keystone species for maintaining the ecosystem. Additionally, there is increasing eutrophication of wetlands and disruption of terrestrial nutrient cycling from excess nitrogen deposition from anthropogenic activities (Greaver *et al.*, 2016; González de Andrés, 2019). There can also be climate-related disruptions to microbial populations, affecting both direct plant–microbe interactions and microbial activities associated with degradation of organic material, fixation of inorganic nutrients, and general nutrient cycling processes (Greaver *et al.*, 2016; Jansson and Hofmockel, 2020). Thus, taking into account the fragility of some of their key growing environments, and the relatively slow growth rates of bryophytes in those environments, studies have estimated that climate change effects on species composition and biodiversity may be particularly acute for bryophytes (He *et al.*, 2016; Becker Scarpitta *et al.*, 2017; Norby *et al.*, 2019; Ruklani *et al.*, 2021). In turn, given that peat bogs contain around a quarter of all terrestrial carbon, it raises concern for changes in global carbon fluxes, as sustained warming of bog ecosystems may cause a major release of carbon to the atmosphere. Additionally, episodic droughts and elevated nitrogen deposition rates due to reduced water retention can also affect net carbon sequestration in *Sphagnum*-dominated vegetation (Limpens *et al.*, 2011). Yet, until recently, comparatively few studies had examined bryophyte communities. Notably, Zanatta *et al.* (2020) estimated that by 2050 only ~30% of current bryophyte species would be in equilibrium within their environments, based on predictive modelling with 40 bryophyte species. As environmental change accelerates, more frequent droughts together with enhanced nutrient turnover due to elevated temperature may favour fast-growing species or those with rapid colonization potential, reducing overall biodiversity.

The specialized metabolite biosynthetic capacity of individual bryophyte species may well influence their ability to adapt to the environmental challenges resulting from global climate change. For example, the potential of various aquatic moss and liverwort species to respond to raised UVB radiation exposure has been examined (Martínez-Abaigar *et al.*, 2006). Hornworts may be of particular interest in this regard, given their apparent lack of the flavonoid pathway. Specialized metabolites may also be components in the biosynthetic pathways that influence how species adjust in response to environmental change. *Sphagnum* species increased phenolic production as a competitive response to vascular plants (Chiapusio *et al.*, 2018), and *Sphagnum* growing in warmer conditions had increased polyphenol concentrations (Sytiuk *et al.*, 2022, Preprint). Moreover, because of their functions in inhibiting tissue degradation, phenolics are a central component in maintaining a stable bog ecology.

## Concluding comments

Phytochemical research over many years has revealed a great diversity of specialized metabolites in bryophytes, with a spectrum of *in vitro* bioactivities. In contrast, there are few studies on either the biosynthetic pathways for these or their functions *in planta*. However, this is rapidly changing, with current advances in our understanding of specialized metabolism in bryophytes. In particular, the recent advent of model species supported by whole genome sequences and molecular technologies for liverworts and mosses, *M. polymorpha* and *P. patens* respectively, has allowed the characterization of biosynthetic genes, regulatory factors and *in planta* functions for specific phenolics and terpenoids. There is now a need for additional model systems that represent more of the diversity of bryophytes, including hornworts and leafy liverworts. Whole genome sequences and transformation protocols have recently been developed for *A. agrestis*, which is proposed as the first hornwort model species (Frangedakis *et al.*, 2021). Significant resources are also being directed into developing molecular tools for studying *Sphagnum* ecosystems that comprise a complex mix of species (Weston *et al.*, 2018). Our increased understanding of specialized metabolism in bryophytes is not only revealing bryophyte-specific compounds and functions, but also furthering our understanding of the evolution and function of specialized metabolite pathways across land plants.
